# Lysophosphatidylinositol Signalling and Metabolic Diseases

**DOI:** 10.3390/metabo6010006

**Published:** 2016-01-15

**Authors:** Syamsul A. Arifin, Marco Falasca

**Affiliations:** 1 Inositide Signalling Group, Centre for Cell Biology and Cutaneous Research, Blizard Institute, Queen Mary University of London, 4 Newark Street, London E1 2AT, UK; s.ahmadarifin@qmul.ac.uk; 2 Department of Basic Medical Science for Nursing, Faculty of Nursing, IIUM, Bandar Indera Mahkota, Kuantan Pahang 25200, Malaysia; 3 Metabolic Signalling Group, School of Biomedical Sciences, Curtin Health Innovation Research Institute Biosciences, Curtin University, Perth 6102, Australia

**Keywords:** lysophosphatidylinositol, G protein-coupled receptor 55, Metabolic diseases

## Abstract

Metabolism is a chemical process used by cells to transform food-derived nutrients, such as proteins, carbohydrates and fats, into chemical and thermal energy. Whenever an alteration of this process occurs, the chemical balance within the cells is impaired and this can affect their growth and response to the environment, leading to the development of a metabolic disease. Metabolic syndrome, a cluster of several metabolic risk factors such as abdominal obesity, insulin resistance, high cholesterol and high blood pressure, and atherogenic dyslipidaemia, is increasingly common in modern society. Metabolic syndrome, as well as other diseases, such as diabetes, obesity, hyperlipidaemia and hypertension, are associated with abnormal lipid metabolism. Cellular lipids are the major component of cell membranes; they represent also a valuable source of energy and therefore play a crucial role for both cellular and physiological energy homeostasis. In this review, we will focus on the physiological and pathophysiological roles of the lysophospholipid mediator lysophosphatidylinositol (LPI) and its receptor G-protein coupled receptor 55 (GPR55) in metabolic diseases. LPI is a bioactive lipid generated by phospholipase A (PLA) family of lipases which is believed to play an important role in several diseases. Indeed LPI can affect various functions such as cell growth, differentiation and motility in a number of cell-types. Recently published data suggest that LPI plays an important role in different physiological and pathological contexts, including a role in metabolism and glucose homeostasis.

## 1. Introduction

Metabolic diseases, such as diabetes, cardiovascular disease and obesity, can be a result of genetics factors, a deficiency in a certain hormone or enzyme, an unbalanced diet or very often a combination of these factors. Obesity, diabetes and cardiovascular disease are strictly interlinked, since obesity is known to increase the risk of cardiovascular disease in adults and to be highly associated with insulin resistance in normo-glycaemic individuals and in patients with type 2 diabetes [1]. Diabetes is the most common metabolic disease and currently one of the major health problems worldwide and it is growing at a very fast rate, mainly because of its strong link with obesity. Diabetes is associated with several complications, including micro and macrovascular complications that are mainly caused by poorly controlled blood glucose levels and that ultimately result in reduced life expectancy. Diabetes can be classified in type 1, an auto-immune disease in which the insulin-producing β cells in the pancreas are destroyed, and type 2, which can be the consequence of acquired environmental agents, as well as genetic factors. Transition from normal glucose tolerance to impaired glucose tolerance and ultimately to type 2 diabetes is accompanied by a decrease in both insulin sensitivity and pancreatic β cell function. Metabolic disorders are often associated to an abnormal increase in dietary fats and the caloric surplus leads to impaired lipid metabolism and fat accumulation which is a fundamental step in the progression of metabolic disorders [2]. Lipids are the major components of cellular membranes and a source of energy and play a crucial role in cellular and physiological energy homeostasis. Lipids include phospholipids and neutral lipids, primarily triacylglycerols and sterol esters. Phospholipids are key components of the membrane structure and major substrates for enzymes, such as phospholipase A (PLA) phospholipase C (PLC), phospholipase D (PLD) and lysophospholipase D. Lysophospholipids are the product of the activity of PLA2 on phospholipids and are well known to act as extracellular signals. Lysophospholipids acting as signalling mediator include lysophosphatidic acid, sphingosine-1-phosphate, lysophosphatidylserine and LPI. In the past two decades lysophospholipids have emerged as second-messenger molecules regulating intracellular signalling pathways that are involved in many physiological and pathological functions, such as inflammation, angiogenesis, nervous system regulation, atherosclerosis and tumorigenesis [3,4,5]. This review will focus on the production and signalling pathways downstream LPI and the emerging evidence suggesting its role in metabolism, with specific interest in LPI, and its main receptor GPR55 involvement in metabolic diseases.

## 2. Lysophosphatidylinositol

LPI is a bioactive lipid generated by the PLA family of lipases which is believed to play an important role in several physiological and pathological processes [6]. In the past thirty year, LPI has been shown to affect various cellular actions such as cell growth, differentiation and motility, in a wide range of cell-types, including cancer cells, endothelial cells and nervous cells. LPI can be the product of the PLA family of lipases comprising PLA_1_ and PLA_2_. PLA_1_ and PLA_2_ enzymes remove fatty acids from the *sn*-1 and *sn*-2 position of glycerophospholipids respectively, generating 2-acyl-lysophospholipids and 1-acyl-lysophospholipids. There are few data on the different biological activity of 2-acyl compared to 1-acyl-LPI, as well as the activity of distinct LPI species containing different fatty acid chains [6]. Recently published data suggest that LPI plays an important role in different physio pathological circumstances, including a role in metabolism and glucose homeostasis [7,8]. The first evidence showing a possible physiological role for LPI came with two papers published in the mid-1980s showing a stimulatory effect of LPI in the release of insulin by pancreatic islets [9,10]. Subsequently, our work clearly identified LPI as a potent mitogenic factor [11,12,13]. Interestingly, our early work identified a synergistic effect of LPI and insulin in promoting cell proliferation on thyroid cells FRTL5 [11]. Importantly, a key role in metabolically active tissues has also been identified [14] and evidence further suggests that LPI is involved in the regulation of fat deposition. Specifically, it has been observed that plasma levels of LPI were significantly higher in obese female patients and positively correlated with weight and percentage body fat [7]. Furthermore, in visceral adipose tissue explants and primary differentiated visceral adipocytes, LPI induces the expression of genes involved in fat deposition.

The LPI biological activities can be divided in non-receptor and receptor-mediated [6]. Recently, the orphan receptor GPR55 has been proposed as potential LPI receptor [15,16]. GPR55 is widely expressed in the brain, especially in the cerebellum as well as in the jejunum and ileum. Importantly, recent studies have demonstrated that GPR55 is also expressed in the endocrine pancreas and in pancreatic β cells where it is involved in stimulus-secretion coupling of insulin secretion, suggesting a role in glucose homeostasis [17]. This would be consistent with the reported effect of LPI on the stimulation of insulin secretion, which has been partly ascribed to its ability to induce intracellular calcium release in rat and mouse pancreatic islets [9,10]. It has been suggested that the LPI-induced insulin release could be correlated with its ability to stimulate the calcium-dependent exocytosis activity, as it has been very recently shown in PC12 neuroendocrine cells [18]. Indeed, exocytosis in PC12 cells is Ca^2+^-dependent, and therefore the increase in intracellular Ca^2+^ induced by LPI could trigger exocytosis. It is currently unknown whether this LPI action is mediated by GPR55 or other receptors, or by its physical properties. However, it is well-known that LPI effect on calcium is mediated by GPR55 in different cells [6]. In addition to acting on GPR55, LPI could also have non-receptor mediated effects. For instance, it has been recently demonstrated a receptor-independent effect of LPI on the Ca^2+^-activated K^+^ channels single-channel activity of endothelial cells [19]. Similarly, it has been shown that LPI activates TREK channels such as bTREK-1 and bKv1.4 K^+^ currents [20]. Furthermore, it has been shown that LPI activates TRPV2 channels in prostate cancer cells [21].

## 3. GPR55

### 3.1. Structure and Distribution

The human GPR55 is composed of 319 amino acids and has an expected molecular weight of 37 kDa and is encoded by the *GPR55* gene located on chromosome 2q27. It was first cloned in 1999 and belongs to the purine cluster of rhodopsin family receptors [22]. It displays sequence similarity to cannabinoid receptors CB1 (13%) and CB2 (14%). Furthermore, it has homologies with other GPCRs such as GPR23 (30%), P2Y5 (29%), GPR35 (27%) and chemokine receptor CCR4 (23%). In human, GPR55 mRNA transcript have been found in the brain regions of caudate and putamen [22], adipose tissue, testis, myometrium, tonsil, adenoid and spleen [23]. In mouse, GPR55 mRNA expression was identified in adrenal, spleen, jejunum, ileum, frontal cortex, hippocampus, cerebellum, dorsal striatum and hypothalamus [17,24]. In addition, diverse range of human cancer cell lines are also expressing GPR55 including ovary, prostate [25], breast [26,27], skin [28], as well as cervix, liver, blood and pancreas [26]. Despite being listed as an orphan receptor in the IUPHAR database, several endogenous and pharmacological ligands have been reported to activate GPR55 [24]. Initially, GPR55 was considered as an atypical cannabinoid receptor (CB) due to its activation shown by ∆^9^-tetrahydrocannabinol, abnormal cannabidiol, and its synthetic derivative, O-1602, as well as by endogenous cannabinoids anandamide, palmitoyl ethanolamine and oleoyl ethanolamine [24]. Interestingly, another paper published in the same year by Oka *et al.* [15], has identified a lysophospholipid, LPI, as the endogenous ligand for GPR55. The potent LPI agonist activity toward GPR55 was also demonstrated by other studies [29,30,31,32]. Recently, a nomenclature review for lysophospholipids receptors considered GPR55 as a provisional LPI receptor with the receptor name LPI_1_ and gene names *LPIR1/Lpir1* for human and non-human genes, respectively [33].

### 3.2. GPR55 Signalling

The pharmacology of GPR55 appears to be much entangled. It is unclear whether this receptor is another member of the CB family or not, due to conflicting data about its activation by endocannabinoids and non-cannabinoid ligands [34]. The sensitivity of GPR55 to endocannabinoids such as anandamide [24] and not to other endocannabinoids [30] makes it a good candidate. On the other hand, its phylogenetically distinction from traditional CB receptor has prevented its classification as a novel CB receptor. However, the weight of evidence point to LPI as the most promising endogenous ligand for GPR55 [15,29,35,36]. The selectivity of LPI as the GPR55 ligand was studied by Kotsikorou *et al.* [37]. They discovered that GPR55 accommodates LPI in the horizontal binding pocket within the transmembrane domain 2 of its polar head group. It has now been demonstrated that GPR55 is associated to Gα12/13 and Gαq subunits and that it can activate several signalling pathways. Upon LPI stimulation of human osteosarcoma cell line U20S, Gαq subunit is able to stimulate PLC activity that induces Ca^2+^ release from the endoplasmic reticulum, activating different PKC isoforms. PKCs catalyse the phosphorylation of different intracellular proteins, such as MAPK and related signalling pathways. GPR55 activation by LPI stimulation was shown to activate ERK1/2 and to be able to activate two transcription factors, such as the cAMP response element-binding protein (CREB) and the nuclear factor kappa-light-chain-enhancer of activated B cells (NF-κB), which can then regulate gene transcription [38]. Moreover, upon LPI stimulation, Gα12/13 activates the RhoA/ROCK signalling pathway. GPR55 activation of RhoA/ROCK signalling pathway regulates PLC, actin cytoskeleton and p38/Activating transcription factor 2 (ATF2) activities [38]. Finally, prolonged oscillatory Ca^2+^ release from intracellular stores leads to the activation and translocation of the nuclear factor of activated T cells (NFAT), which can then regulate DNA transcription and gene expression [29]. Both MAPK and RhoA/ROCK signalling pathways are able to control and regulate a plethora of cellular functions, such as cell proliferation, cell division, apoptosis, cell differentiation and actin cytoskeleton remodelling. Moreover, MAPKs and RhoA/ROCK signalling pathways were found to be deregulated in various cancers and several studies suggest the use of inhibitors of these pathways to counteract cancer cell growth and metastatic development [39].

## 4. LPI/GPR55 Signalling and Physiological Function

### 4.1. Metabolism

There are increasing reports that GPR55 may play a role in the modulation of energy homeostasis. Initial indication of the possible involvement of GPR55 in metabolism, was provided by the evidence that LPI induced insulin release from pancreatic islets via mobilisation of Ca^2+^ ion [9,10]. Consistent with this, a study on isolated islet has revealed high GPR55 mRNA expression and its activation with pharmacological agonist O-1602 showed an increase in Ca^2+^ release and insulin secretion stimulated by glucose [17]. The *in vivo* data from the latter study also showed that GPR55 activation increases glucose tolerance and plasma insulin levels. Furthermore, similar results were obtained by another study on isolated islet stimulated with various GPR55 agonists. It was shown that GPR55 agonists such as Abn-CBD PEA, OEA, O-1602, and AM-251, significantly stimulate insulin secretion and later confirmed to lower blood glucose in *in vivo* studies [40].

### 4.2. Endothelial Cells and Vasculature

Increasing evidence supports an endothelial site of action for LPI and suggests a possible role for LPI/GPR55 axis in vasculature. For instance, studies suggested that GPR55 relaxes rat mesenteric resistance artery when activated by LPI and not by atypical cannabinoids. LPI, indeed, induces calcium release in rat mesenteric artery endothelial cells with vasodilator effects [41,42]. LPI is an endothelium-dependent vasodilator in rat small mesenteric artery and a hypotensive agent. Signalling pathways activated by LPI in endothelial cells include PLC-IP3 and ROCK-RhoA to elevate intracellular Ca^2+^. Studies using the selective antagonist CID16020046 uncovered a potential role for G protein-coupled receptor 55 in platelet and endothelial cell function [43]. It has been shown that CID16020046 reduced LPI-induced wound healing in primary human lung microvascular endothelial cells and impeded LPI-inhibited platelet aggregation, suggesting a novel role for LPI/GPR55 axis in platelet and endothelial cell function [43].

### 4.3. Gastrointestinal Functions

The interest on bioactive phospholipids present in the gastrointestinal mucosa has rapidly grown during the past decade. Recently, it has become increasingly evident that lysophospholipids can influence gastrointestinal functions and ultimately hormones and peptides release from the gut. Indeed, it has been shown that food-derived lipids can directly modulate gastrointestinal functions. In addition, it has been observed that the digestive tract itself is able to release different types of bioactive lipids, together with polypeptide hormones, into the lumen. GPR55 has been found to be largely expressed in the gastrointestinal tract and therefore several studies have focused their attention on the potential pathophysiological role of GPR55 in gastrointestinal functions [44]. It has been suggested that GPR55 may have a role in intestinal inflammation [44]. Indeed, the GPR55 antagonist CID16020046 has been shown to have anti-inflammatory activity in mice subjected to different models of intestinal inflammation [45]. It has been recently shown that postprandial plasma phospholipids in men are influenced by the source of dietary fat [46]. In particular, postprandial elevation of LPI has been found after dairy and soy meals with important implications for LPI effect on metabolic processes. Interestingly, it has been recently demonstrated that LPI can also activate GPR119 in RH7777 rat hepatoma cells stably expressing human GPR119 [47]. GPR119 is expressed predominantly in the pancreas and gastrointestinal tract and its activation has been shown to cause a reduction in food intake and body weight gain in animal models [48]. In addition, it has been shown that the activation of GPR119 can induce incretins release in the intestine. However, GPR119 appears to be activated by other lipids, including lysophospholipids, with higher affinity compared to LPI and therefore further studies are required in order to assess if, in particular conditions, GPR119 may mediate LPI effects *in vivo*.

### 4.4. Central and Peripheral Nervous System

GPR55 mRNA is expressed in microglial from primary mouse, BV-2 cell line [49] and neurons [50]. It has been suggested that LPI activated GPR55 is able to induce exocytosis and catecholamine release from the neuroendocrine pheochromocytoma-12 cells [18,35] Furthermore, the activation of dorsal root ganglion (DRG) neurons by GPR55 increases intracellular calcium and inhibits potassium current through M-type potassium channel [30]. This neuronal excitability of GPR55, particularly on large diameter DRG neurons, suggested its pro-nociceptive role [30]. The hypothesis that GPR55 activation inhibits neurogenic contractions in the gut has been recently investigated. By testing the inhibitory effect of the atypical cannabinoid O-1602, a GPR55 agonist, in mouse colon, it has been found that the activation of GPR55 leads to inhibition of neurogenic contractions in the gut [51]. Noteworthy, chronic intake of high fat and obesogenic diet has resulted in a significant reduction in the response of intestinal afferents to satiety mediators such as cholecystokinin and 5HT [52]. The authors of the latter study suggested that the impairment of vagal afferent function involves decreased satiety signalling from gastrointestinal organs to the brain [52]. Consistently with this, diminished vagal nerve signalling in obesity could lead to enhanced inflammation and metabolic complications [53].

### 4.5. Nociception and Inflammation

Several studies have implicated GPR55 in neuropathic and inflammatory pain. In two models of hyperalgesia, it has been reported that inflammatory mechanical hyperalgesia induced by administration of Freund’s complete adjuvant was completely absent in GPR55 knockout mice [54]. A similar response is obtained when GPR55^−/−^ mice undergo partial nerve ligation [55], an established pre-clinical model for neuropathic hyperalgesia. Moreover, the authors demonstrated that the levels of the anti-inflammatory cytokines Interleukin-4, Interleukin-10, Interferon γ and Granulocyte Macrophage-Colony Stimulating Factor, were higher in GPR55^−/−^ mice compared to control mice 14 days post FCA-injection [54]. A recent study may provide an explanation for the absence of hyperalgesia in GPR55^−/−^ mice [50]. The authors identified the GPR55 involvement in central pain processing and unravelled a novel Ca^2+^-mediated mechanism through activation of periaqueductal grey neurons by GPR55 [50]. Consistently, new data suggested a possible role of GPR55 in the inflammatory system. Indeed, GPR55 is overexpressed in monocytes and NK cells and its activation using a synthetic agonist (O-1602) is able to stimulate IL-2, IL-12, and TNFα. Moreover, the activation also increases CD69 activation marker expression, granzyme B, CD107-dependent cytotoxicity and IFN-γ, suggesting a pro-inflammatory role of GPR55 in the innate immunity [56].

### 4.6. Bone

Whyte and colleagues have found that GPR55 agonist O-1602, affects the regulation of bone mass (osteoclastogenesis), stimulating osteoclast resorption, suppressing formation *in vitro* and reducing bone resorption *in vivo* in mice [57]. They also reported an increased GPR55 expression in osteoclasts, compared to macrophages precursor. Consistently with this, activation of CB2 and the inhibition of GPR55 by cannabidiol promotes migration of mesenchymal stem cells in the regenerative process of bone healing [58].

## 5. LPI/GPR55 Axis and Disease

### 5.1. Obesity and Type-2 Diabetes

The imbalance of energy homeostasis could lead to metabolic disorders such as obesity and type 2 diabetes. It was reported that a polymorphism in Val195 allele of GPR55 gene has a link with anorexia nervosa, a disorder characterised by low body weight and obsessive fear of weight gain [59]. This report was later confirmed by a study in humans that discovered higher levels of GPR55 expression in visceral adipose tissue and sub-cutaneous tissue correlated with an increase in body weight and fat percentage [7]. In addition, another study also showed that O-1602 influences obesity by acutely stimulate food intake and chronically increases adiposity in Sprague-Dawley rats [60]. Imbernon *et al.* (2014) compared the expression of GPR55 and CB1 receptors expression in rat white adipose tissue and discovered increased levels of the GPR55 expression after fasting and recovered after leptin treatment [8]. However, GPR55 expression showed an increase throughout 90 days of the experiment and only reduced during the gestation period. Recently, Meadows *et al.* (2015), reported significant increase in fat mass and insulin resistance in GPR55^−/−^ mice [61]. They also discover that locomotor and voluntary physical activity substantially reduced with intact muscle function, thus suggesting that the selective decrease in physical activity is caused by GPR55 ablation and not due to changing of feeding behaviour as CB_1_. However, the fact that GPR55 is expressed in enteric neurons could suggest that GPR55 has a role in gastrointestinal functions, such as secretion and motility [62]. In addition, LPI has been found to counteract the symptoms of diabetes such as high blood glucose, lower body weight, increase amplitude of slow wave, and to improve gastric emptying in streptozotocin induced diabetes in rat. The authors also discover that GPR55 mRNA is up regulated in the STZ-induced diabetic condition. Furthermore, treatment of the diabetic rats with LPI induces increased levels of motilin, gastrin, vasoactive intestinal peptide and somatostatin, indicating the role of GPR55 in controlling movement in the gut [63]. Recently, the functional role of GPR55 in diabetes-induced nephropathy was studied in rat proximal tubules. It was shown that GPR55 mRNA and protein expression in proximal tubule has increased in response to high glucose in presence or absence of high albumin [64]. The authors suggest that GPR55 may play a functional role in the tubules that is altered in response to pathophysiological conditions. A potential role for LPI in type-1 diabetes pathogenesis has been recently suggested. Indeed, a lipidomic characterization of a genetically modified mouse model of the early stages of human type-1 diabetes revealed an increase in LPI in the pre-type 1 diabetic mouse compared to control mice. These findings open an intriguing scenario for LPI suggesting that LPI may play a role in autoimmune diseases and inflammation [65].

### 5.2. Cardiovascular Disease

A recent study has suggested that GPR55 is able to control the adrenergic signalling pathway in the heart and to have a potential role in the pathogenesis of heart failure [66]. Interestingly, the cardiac function of young GPR55 knockout mice was the same as control mice, whereas mature GPR55 knockout mice were characterised by systolic dysfunction and ventricular remodelling compared to control mice [66]. Recently, LPI has been found to increase significantly following asphyxia-induced cardiac arrest using a rat model [67]. Interestingly, in the same rat model when cardiac arrest was followed by cardiopulmonary resuscitation the LPI levels decrease drastically. These data suggest that LPI levels could be regulated by cellular conditions associated with ischemia and reperfusion and have possible important implications for improving atherosclerosis. Interestingly, measurements of the levels of lysophospholipids in 141 patients undergoing coronary angiography revealed that lysophospholipids, including LPI, were elevated in acute coronary syndrome patients [68]. Furthermore, a study on human macrophages and foam cells revealed that activation of GPR55 by O-1602 exacerbates oxidative low-density lipoprotein-induced lipid accumulation and inflammatory responses, reducing cholesterol efflux [69].

### 5.3. Cancer

Several studies highlighted the role of GPR55 in several types of cancer and we have recently reviewed the role of the LPI/GPR55 axis in cancer [70]. Our group demonstrated that GPR55 has a key role in cancer by defining a new autocrine loop that involves GPR55, LPI and the ABC transporter, ABCC1, in prostate and ovarian cancer cell lines [71]. Another report revealed that this receptor plays an important role also in the modulation of migration and invasion of human breast cancer cell lines [27]. Furthermore, another study demonstrated that downregulation of GPR55 in a xenograft mice model of glioblastoma reduces tumour growth [26] and that GPR55 knockout mice are more resistant to skin carcinogenesis [28].

## 6. Conclusions

**Figure 1:**
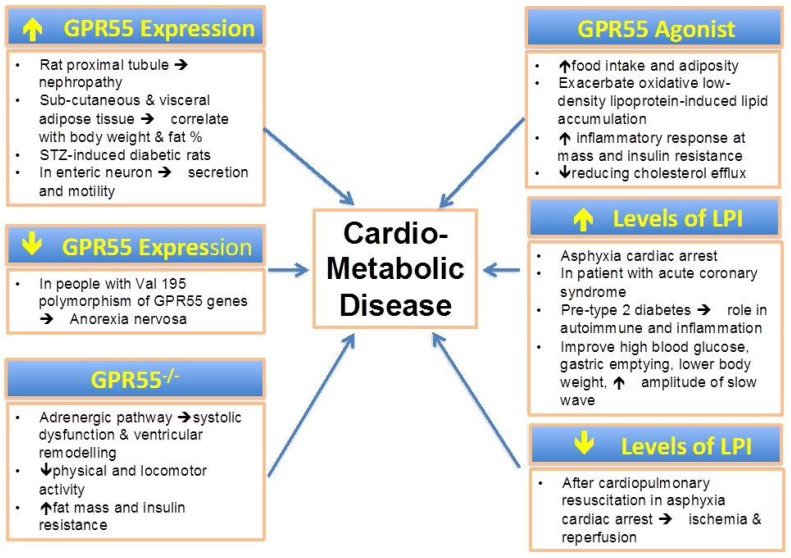
The pathophysiological relevance of GPR55 expression, levels of LPI and potential role of its agonist in cardio-metabolic diseases.

Increasing evidence suggest that LPI play a key role in several metabolic functions and possibly in metabolic disorders (Figure 1). In parallel, GPR55, the main LPI receptor, has been proposed as a metabolic regulator [72,73]. The LPI/GPR55 axis has been shown to be positively associated with obesity in human. Altered levels of LPI have been found in obesity and diabetes. In addition, the LPI/GPR55 system is also involved in inflammation and cancer. Further mechanistic studies are required to elucidate the potential role of LPI in the pathogenesis of metabolic disorders.
